# Concomitant intensive chemoradiotherapy induction in non-metastatic inflammatory breast cancer: long-term follow-up

**DOI:** 10.1038/sj.bjc.6603987

**Published:** 2007-09-18

**Authors:** D Genet, C Lejeune, P Bonnier, Y Aubard, L Venat-Bouvet, D J Adjadj, J Martin, J L Labourey, A Benyoub, P Clavère, V Lebrun-Ly, P Juin, L Piana, N Tubiana-Mathieu

**Affiliations:** 1 Department of medical oncology, CHU Dupuytren, Limoges, France; 2 Department of medical oncology, CHU de la Conception, Marseille, France

**Keywords:** inflammatory breast cancer, chemoradiotherapy, breast conservation

## Abstract

The aim of this study was to evaluate with a long follow-up the efficacy of concomitant chemoradiotherapy in non-metastatic inflammatory breast cancer (IBC) and to evaluate the breast conservation rate. Between 1990 and 2000, 66 non-metastatic patients with IBC were treated with chemotherapy and concomitant irradiation. The induction chemotherapy consisted of epirubicine, cyclophosphamide and vindesine, in association with split-course bi-fractionated irradiation to a total dose of 65 Gy with concomitant cisplatin and 5-fluorouracil. Maintenance chemotherapy consisted of high-dose methotrexate and six cycles of epirubicine, cyclophosphamide and fluorouracil. Hormonal treatment was given if indicated. Mastectomy was not systemic. Among 65 evaluable patients, 57 (87.6%) achieved a complete clinical response and had a breast conservation. Only six loco regional relapses were noted in six patients with a delay of 20 months and with concomitant metastatic dissemination in four cases. Median disease-free survival (DFS) was 28 months. Median overall survival (OS) was 63 months and median follow-up was 55.5 months. Induction chemotherapy and concomitant irradiation is feasible in patients with IBC, permitting a breast conservation with a high rate of local control with an OS comparable to that of the best recent series.

Inflammatory breast cancer (IBC) accounts for 1–6% of all invasive breast tumours (Chang *et al*, 1998). Its incidence has doubled over the last 20 years (Hance *et al*, 2005).

Despite advances in treatment with induction chemotherapy regimens, disease-free survival (DFS) and overall survival (OS) remain unsatisfactory with a 30–50% 5-year survival in the largest studies (Anderson *et al*, 2003; Cristofanilli *et al*, 2003; Somlo *et al*, 2004; Lerebours *et al*, 2005). The role of surgery remains controversial even though it is often used in protocols (Schafer *et al*, 1987; De Boer *et al*, 2000). The achievement of pathologic response to induction chemotherapy is an excellent indicator of prolonged (DFS) and (OS) (Ueno *et al*, 1997). This is achieved in less than 30% of patients (Chevallier *et al*, 1993).

Based on these results, we conducted a protocol combining the most active drugs such as anthracycline with radiotherapy as the major local treatment as the initial treatment without mastectomy.

We report the long-term efficacy of this protocol in 66 patients with non-metastatic IBC.

## MATERIALS AND METHODS

Between 1990 and 2000, 66 consecutive patients with non-metastatic IBC received treatment. All the patients were examined by a multidisciplinary team and gave their written informed consent for participation. Eligibility included clinical and/or pathological criteria for IBC as defined by Haagensen (Haagensen, 1971). Non-metastatic IBC was defined by negative thoraco-abdominal scan and bone scan.

**Figure 1:**
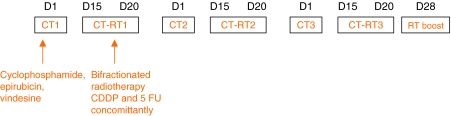
Induction treatment protocol plan. Three Cycles with 28 days delay.

The protocol was described previously (Tubiana-Mathieu *et al*, 2001) as shown in Figure 1: three cycles with a 28-day delay associated in day 1, epirubicine (E) (50 mg m^−2^), cyclophosphamide (C) (350 mg m^−2^) and vindesine (V) (2 mg m^−2^), and CDDP (10 mg m^−2^ day^−1^), 5-FU (500 mg m^−2^ day^−1^) in days 15–20 concomitantly with bifractioned radiotherapy. After the first 24 patients, the doses of chemotherapy were increased as follows: C 500 mg m^−2^, E 75 mg m^−2^ and CDDP 20 mg m^−2^ days 15–20. Radiotherapy was performed as split course to a dose of 45 Gy with a twice daily schedule of 15 Gy for each course in 10 fractions for 5 days (1.5 Gy per fraction). The three series were performed from day 15 to 20 of each induction course concomitantly with CDDP and 5-FU. The volume included the whole breast by two tangential portals and supra clavicular, internal mammary chain, and eventually axillary lymph nodes by direct portal (axillary lymph nodes were not radiated after axillary dissection).

All volumes were treated by a ^60^Co or six megavolt photon beam except the internal mammary chain, which was treated by a mixed 50–60% beam with photon and electron beam of adequate energy.

A boost irradiation course was performed 8 days after the 3rd series. Twenty-four to 26 Gy (calculated as ICRU criteria) in 12–13 daily fractions schedule over 16–17 days were administered to the entire breast, including the skin, but not the rib cage. This boost course was given without chemotherapy.

Methylprednisolone (60 mg D^−1^ po) and Warfarin (1 mg D^−1^ po) were given for the first 3 months because 30% of the initial 20 patients developed upper limb thrombophlebitis. All cycles were supported by granulocyte colony stimulating factor given from day 3 to 9 of each cycle.

After the radio chemotherapy programme, patients received three cycles of high-dose methotrexate (MTX) (1.5 g m^−2^) every 15 days and six cycles of FEC 50 (5-FU 500 mg m^−2^, E 50 mg m^−2^ and C 500 mg m^−2^) every 21 days as maintenance chemotherapy.

Patients with ER and/or PR positive breast cancer received hormonotherapy: tamoxifen 20 mg day^−1^ for 5 years in pre- and postmenopausal patients after the end of chemotherapy and with analogue LH-RH in premenopausal patients.

The clinical response was evaluated after each cycle of the induction treatment. Multiple breast biopsies were performed after the induction from 1994.

Surgery (mastectomy or quadrantectomy) was performed only in case of stable disease or partial clinical or pathologic response.

After completion of the treatment, patients underwent physical examination at least once every 4 months for the first 2 years and every 6 months for 5 years. A yearly mammogram was performed, with bone scans and chest X-rays only according to symptoms.

## STATISTICAL METHODS

The primary end point of this study was clinical tumour response defined in 1990 as follows: a complete response (CR) was defined as the complete disappearance of all clinical evidence of disease; partial response (PR) corresponded to a greater than 50% decrease in tumour area; no change (NC) was less change than partial regression without progressive disease; and progressive disease (PD) corresponded to 25% or more increase in tumour area or increased nodal stage or both.

Pathological response was assessed by six true-cut biopsies performed after chemoradiotherapy. Pathological response was complete (pCR) with no residual tumour cell on all biopsies.

Overall survival and DFS were calculated from diagnosis to last contact for living patients. Follow-up was calculated with the same variables. Disease-free survival was defined as the time of any type of recurrence or death of any cause. The standard Kaplan Meier method was applied for survival analysis.

Toxicities of treatment were graded according to the WHO scale.

## RESULTS

### Patients’ characteristics

Median age was 48 years (25–70). Thirty-four were pre-menopausal and 32 postmenopausal.

The initial biopsy of the breast and the skin performed in all patients found 53 ductal, 11 lobular and two undifferentiated carcinoma. Sixty-two had clinical IBC with dermal lymphatic involvement in 43 patients. Four had occult disease, all with dermal lymphatic involvement.

To limit the radiotherapy fields, an axillary surgery was performed in the first 45 patients at the same time of indwelling catheter placement. Nodes picking were performed in nine patients, with 1–5 nodes being removed, and all were involved. Thirty-six axillary dissections were performed, with a median of 15 nodes being removed (8–32); 84% showed 7 or more involved nodes.

Hormone receptor status was known in 58 patients. Estrogen/progesterone receptors were positive/positive in 19 patients, negative/negative in 28, and positive/negative in 11.

### Evaluation of response

The clinical response was evaluated in 65 patients; one was not evaluated because of death from pulmonary embolism at day 15.

Fifty-seven patients (87.6%) achieved clinical CR, seven (11%) a clinical PR, and one a progression disease on the skin.

CR was obtained after two courses in 13 patients (38%) and after three courses in 44 patients (62%).

Pathological response was assessed in 25 patients: 22 had a pathologic complete response (pCR).

Among the seven patients with a partial clinical response, mastectomy was performed in five patients: in two patients, viable cells were not found in the breast (these two patients had a long time survival of 51 and 80 months without relapse) and in three patients, tumour viable cells were found (two patients developed metastasis and one had no relapse after surgery). For the other two patients, mastectomy was not performed due to the dramatic evolution: one developed brain metastases at 5 months and died at 7 months and the other after tumorectomy developed pulmonary metastasis at 6 months and died at 16 months.

### Evaluation of survival

The median follow-up of the 66 patients was 55.5 months (range 4–178) in all patients and 89 months (28–178) in surviving patients.

**Figure 2:**
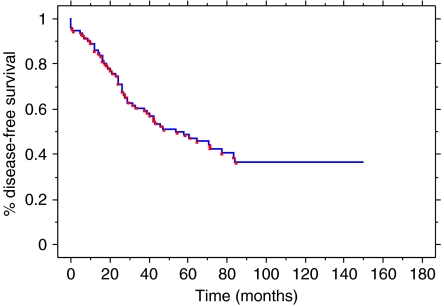
Disease-free survival for all patients.

**Figure 3:**
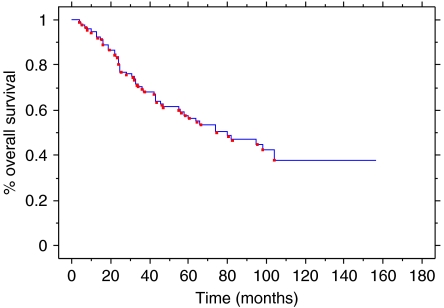
Overall Survival for all patients.

Among the 66 patients, 37 deaths occurred. All but four were related to breast cancer (the four non-related deaths were pulmonary embolism at 3 months, acute lymphoblastic leukaemia at 13 months, heart failure at 28 months and cirrhosis at 75 months). Median OS was 63 months and median DFS was 28 months. The 5- and 10-year OS were 50.3 and 38%, respectively (Figures 2 and 3).

### Pattern of failure

As shown in Table 1, six patients developed local recurrence after a median delay of 20 months (range 7–42); two recurrences were only local without any distant metastasis and were alive without other recurrence at 56 and 89 months, and for the other four patients, local recurrence was associated with metastatic evolution. Five patients presented contralateral disease, with a median delay of 26 months (range 6–50). Two of them were isolated and three in association with distant metastasis. Thirty-six showed a systemic recurrence (seven patients had distant metastasis and local or contralateral recurrence), with a median delay of 19.5 months (1–84). Sites of metastases were principally liver (19 patients), bone (11 patients), lung (6 patients) and brain (6 patients).

### Treatment toxicities

As shown in Tables 2A and 2B, the toxicity of chemotherapy during the induction phase was mainly haematologic with grade 3–4 neutropaenia in 33 patients (50%) and febrile neutropaenia in 8 patients (12%) without septic death. Grade 3–4 thrombopaenia occurred in 16 patients (24%) and grade 3–4 anaemia occurred in three patients (5%). During the maintenance phase, grade 3–4 neutropaenia occurred in 13 patients and grade 3–4 thrombopaenia occurred in 2 patients. Thrombosis occurred in eight patients (five cases occurred before the initiation of the treatment with warfarin; despite this preventive measure, two further cases occurred, one of which had the pulmonary embolism previously cited while receiving warfarin and then heparin). Other toxicities: two patients developed grade 3 serum transaminase elevation, two had grade 2 paraesthesias induced by cisplatin and two had grade 3 mucositis.

Radiotherapy was well tolerated during induction phase. One skin toxicity in an obese patient was noted with a local abscess 10 months after the end of treatment. In six patients, the principal side effect was rib fractures that occurred with a median delay of 12 months after the treatment.

One cardiac injury-related death was noted 31 months after diagnosis. The patient had been treated in childhood for an acute lymphoblastic leukaemia but had a normal cardiac function before breast cancer treatment.

### Feasibility

Fifty-five (83%) patients received 100% of the induction doses, 47 (71%) patients received 100% of the MTX dose, and 47 (71%) patients received 100% of the maintenance FEC 50 dose. Among the 41 patients who received higher doses of chemotherapy, 35 (85%), 27 (65%) and 29 (70%), respectively, received 100% of induction, MTX and maintenance doses. The dose of anthracycline was never decreased. All patients received the protocol radiation dose. The median duration of radiation therapy was 81 days (range 60–98), compared with 70 days as initially planned. The median duration of treatment was 9 months (range 2–13).

## DISCUSSION

This study, with a long follow-up of 55.5 months, had demonstrated the role of intensive initial chemoradiotherapy to improve local control in IBC and to avoid mastectomy with a long survival.

Our population included very homogeneous patients.

The best outcome for these patients classically uses a multimodality therapy, including neoadjuvant chemotherapy, surgery, adjuvant chemotherapy, radiotherapy and endocrine therapy in case of positive receptors.

Radiotherapy was the mainstay of care for IBC. In a trial published in 1987, Schafer *et al* (1987) demonstrated that patients treated with neoadjuvant chemotherapy, surgery and adjuvant chemotherapy but without radiotherapy developed local recurrences 8–17 months after mastectomy. In the present trial, we used an early split course twice daily radiation therapy in combination with chemotherapy. The treatment with a bifractionated irradiation technique, permitting the use of a condensed tissue frame, may improve locoregional control by diminishing tumour cell regeneration between irradiation treatments (Thames *et al*, 1983). An early report of the MD Anderson Cancer Center suggested the potential benefits of using hyperfractionated radiotherapy that were confirmed in a later study (Barker *et al*, 1980; Liao *et al*, 2000). In a previous study, bifractionated irradiation was used after three cycles of chemotherapy and gave a very good rate of local control of 72% (Arriagada *et al*, 1990).

The timing of radiotherapy varied in the different studies: current practices delivered radiotherapy after chemotherapy and do not use it to obtain a rapid response rate.

In the literature, the role of surgery in IBC has been controversial. In the only prospective randomised study comparing surgery *vs* radiotherapy following neoadjuvant chemotherapy in 57 patients with IBC, Mourali *et al* (1993) did not find any significant difference in DFS between the two groups. De Boer *et al* (2000) didn’t find any difference in OS, DFS and disease-free relapse in a retrospective comparison between two groups of IBC patients: one treated by surgery and radiotherapy and the other by radiotherapy alone after neoadjuvant chemotherapy. But patients with complete clinical response were encouraged to have radiotherapy alone. In the trial of MD Anderson (Fleming *et al*, 1997; Ueno *et al*, 1997) in 178 patients, the authors found that patients who had response to induction chemotherapy benefited from the addition of mastectomy to chemotherapy and radiation. A recent retrospective analysis of 485 IBC patients has suggested that mastectomy in association with chemotherapy and radiotherapy seemed to enhance locoregional control with no impact on survival, but patients without mastectomy had more extensive disease at presentation. In our study with radio-chemotherapy, we achieved a high clinical control rate without mastectomy (88%), which is confirmed by the long follow-up of 55.5 months with only six local recurrences in patients in complete clinical response (four with concomitant metastatic evolution) occurring in a median delay of 20 months.

Many different regimens of chemotherapy have been used, but anthracycline based chemotherapy remains the reference of systemic treatment (Chevallier *et al*, 1993; Mourali *et al*, 1993; Bauer *et al*, 1995).

In their retrospective study of 308 patients, Panades *et al*, 2005 found that a more intense chemotherapy regimen improved 10 years breast cancer specific survival. Other series have found no improvement on outcome with intensification (Therasse *et al*, 2003). In our protocol, after 24 patients we intensified the dose of induction chemotherapy as described, and no difference in local response was found before and after this intensification. On the other hand, we were unable to statistically assess the impact of chemotherapy dose on survival. Methotrexate was used after induction treatment for its good meningeal diffusion, but six brain or meningitis metastases occurred. Likewise, the role of maintenance chemotherapy must be elucidated.

The published survival data were obtained in historic trials or in retrospective studies based on single institution experience and have reported that at least one-third of patients treated with multi modality therapy are alive at 5 years. A recent report of the National Cancer Institute based on epidemiologic survey between 1988–1990 and 1997–1999 showed a poor median survival in IBC with 2.9 years (Hance *et al*, 2005). A larger retrospective trial reports the experience of the MD Anderson Cancer Center with a long follow-up of 7.4 years in 178 IBC patients treated with different multimodality protocols during a 20-year period: the median survival was 37 months for all patients. Overall survival at 5 and 10 years was 40 and 33%, respectively. Other studies reporting a long follow-up for IBC achieved an OS at 5 years from 34 to 75% (Liao *et al*, 2000; Harris *et al*, 2003; Baldini *et al*, 2004; Liauw *et al*, 2004), at 10 years range, respectively, from 13 to 47% (Low *et al*, 2004; Cariati *et al*, 2005) and a median OS between 25 to 62 months.

Many questions are not resolved in IBC treatment, such as the contribution of new drugs like Taxanes, the duration of chemotherapy, the best local treatment and the role of new molecular markers. However, the results obtained with initial dose dense chemotherapy and concomitant irradiation permit the proposal of this association as a useful therapeutic option.

## Figures and Tables

**Table 1:** Pattern of failure

**Pattern of failure**	**Number**	**Median delay in months (range)**
*Local recurrence*	6	20 (7–42)
Alone	2	
With systemic recurrence	4	
*Controlateral disease*	5	26 (6–50)
Alone	2	
With systemic recurrence	3	
*Systemic recurrence*	36	19.5 (1–84)

**Table 2A:** Induction haematological toxicity (worst toxicity for any course)

	**G III–IV (%)**
Anaemia	3 (5%)
Neutropaenia	33 (50%)
Febrile neutropaenia	8 (12%)
Thrombocytopaenia	16 (24%)

**Table 2B:** Induction non-haematological toxicity

Nausea vomiting	4 (3 G II, 1 G II)
Neuropathy	3 (G II)
Phlebitis	8
Mucositis	2 (G III)
Rib fracture	6
Serum transaminase	7 (3 G II, 4 G III)
